# Recent advances in graft-versus-host disease

**DOI:** 10.12703/r/12-4

**Published:** 2023-03-06

**Authors:** Aisling M Flinn, Andrew R Gennery

**Affiliations:** 1 Translational and Clinical Research Institute, Newcastle University, Newcastle upon Tyne, NE1 7RU, UK; 2 Great North Children’s Hospital, Newcastle upon Tyne, UK

**Keywords:** Acute graft-versus-host disease, chronic graft-versus-host disease

## Abstract

Acute and chronic graft-versus-host disease (GVHD) continue to present a significant challenge to physicians, accounting for considerable haematopoietic stem cell transplant (HSCT)-related morbidity and mortality, particularly those patients with steroid-refractory disease.

In this review, we discuss recent advances in understanding the underlying pathophysiology, prevention and management of acute and chronic GVHD. Barriers to progress include the difficulty in obtaining high-quality evidence with sufficient patient numbers to identify optimal preventative and treatment strategies, with the heterogeneity of multiple patient, donor, graft and transplant-related factors, in addition to limited availability of human tissue to study the underlying pathophysiology, particularly in steroid-refractory disease. Continued collaborative efforts to improve our understanding of the pathophysiology involved, particularly in steroid-refractory disease, identification of biomarkers to permit risk stratification, and further well-designed randomised clinical trials are essential to help physicians determine optimal GVHD preventative and treatment strategies for each individual patient.

## Introduction

Despite significant advances in many areas of haematopoietic stem cell transplant (HSCT) over the last few decades, graft-versus-host disease (GVHD) continues to account for considerable morbidity and mortality. Acute (aGVHD) and chronic (cGVHD) GVHD encompass two different entities with distinct underlying pathophysiologies. However, strategies to prevent and treat aGVHD and cGVHD often overlap. aGVHD typically manifests in the first 3 months post-HSCT as a characteristic rash, secretory diarrhoea, cholestatic liver dysfunction or a combination of these. In addition to the skin, gastrointestinal tract and liver, targets in aGVHD include the thymus gland and endothelium; however, how to clinically stage/grade their involvement has not yet been established^1,2^. cGVHD typically manifests after HSCT and can affect almost any organ system. An overlap syndrome between the two is well recognised, as is late presentation of aGVHD. In this review, we discuss recent advances in understanding the underlying pathophysiology, prevention and management of aGVHD and cGVHD.

## Pathophysiology

Multiple clinical risk factors for aGVHD, including donor-recipient human leukocyte antigen (HLA) disparity, older patient age, conditioning with total body irradiation or high-intensity chemotherapy, and female donor-to-male recipient, have been identified. The pathophysiology underlying aGVHD involves a sequential inflammatory cascade precipitated by initial inflammatory stimuli, including pre-transplant infection and chemo- and/or radio-therapy conditioning with subsequent activation of the immune system by damage-associated molecular patterns (DAMPs) and pathogen-associated molecular patterns (PAMPs) causing a ‘cytokine storm’ and providing an environment that promotes T-lymphocyte migration to sites of tissue damage, activation and proliferation. Alloreactive T-lymphocyte interaction with antigen-presenting cells (APCs) expressing host peptides leads to the initial activation of the T-lymphocyte (cell) receptor (TCR). In addition to cytokine stimulation and APC interaction, TCR co-stimulation is required; positive and negative co-stimulatory mediators studied in aGVHD models include CD28, ICOS, CD40L, CTLA4 and PD1/PD-L1^3^. T-lymphocyte interaction with APCs and subsequent TCR stimulation triggers the activation of tyrosine kinases and a series of intra-cytoplasmic events culminating in the activation of transcription factors, including NF-kB, NFAT and JNK, leading to T-lymphocyte activation and proliferation. This generates an effector T-lymphocyte response leading to tissue damage directly by cellular cytotoxicity and indirectly via the release of further pro-inflammatory mediators, including tumour necrosis factor-alpha (TNF-α), interferon-gamma (IFN-γ) and interleukin 1 (IL-1). Recent studies have investigated intrinsic players that attempt to counteract this inflammatory response, including the role of regulatory T-lymphocytes (Tregs), innate lymphoid cells and invariant natural killer T-lymphocytes (iNKTs)^4–6^, as well as identifying new areas involved in promoting the inflammatory response, including the NOTCH pathway, Janus kinase (JAK) signalling and Th17 cells^7–9^. Advances in understanding the role of these cell populations and pathways offer potential for clinical translation. The metabolomics employed in this dysregulated immune process has been the subject of recent investigation. Pathways shown to contribute to aGVHD include aerobic glycolysis, oxidative phosphorylation, glutaminolysis and fatty acid oxidation, and understanding these pathways may provide new therapeutic targets^10^. Involvement of the gut microbiome in the pathogenesis of aGVHD has also been the subject of recent investigation. Gut dysbiosis is associated with an increased risk of aGVHD due to disruption of normal gut immune cells and reduction in bacteria which produce short-chain fatty acids, an important nutritional source for intestinal epithelial cells^11^. In addition, microbial metabolites play an important role in maintaining the integrity of the epithelial barrier, and their deficiency contributes to epithelial leakiness, pathogen translocation and immune system activation^12^.

Previously, steroid-refractory aGVHD (SR-aGVHD) has been assumed to reflect continued T-lymphocyte alloreactivity unrestrained by corticosteroids, but more recent studies suggest that specific pathophysiological mechanisms, including endothelial dysfunction, impaired recipient tissue repair and immune tolerance responses, are at play^13,14^. Elevated endothelial stress markers observed in SR-aGVHD include angiopoietin-2, IL-8, soluble thrombomodulin and hepatocyte growth factor^15,16^. Novel transcriptomic findings include increased *CHI3L1* and decreased *AQP8* gene expression in the gut mucosa of patients with gastrointestinal SR-aGVHD^13^, and further studies are needed to understand the role of these genes.

The pathophysiology of cGVHD is less well understood. Contributing factors thought to be involved include thymic damage with disruption to central tolerance causing dysregulated T-lymphocyte development, along with dysregulated peripheral tolerance mechanisms, including dysregulated B-lymphocyte immunity, and chronic inflammation and tissue damage with aberrant tissue repair mechanisms (e.g., due to the release of growth factors such as transforming growth factor-beta [TGFβ] by macrophages leading to activated fibroblasts, collagen deposition and fibrosis).

## Prevention of graft-versus-host disease

Calcineurin inhibitor (CNI)-based GVHD prophylaxis has been the longstanding standard of care in matched sibling and unrelated donor HSCT, in combination with either methotrexate (MTX) or mycophenolate mofetil (MMF) (Table 1). Previous studies have not demonstrated a significant superiority in combinations of ciclosporin/tacrolimus (CSA/TAC) and MTX/MMF, although less toxicity is seen with MMF^17,18^. Sirolimus, a mammalian target of rapamycin (mTOR) inhibitor, is thought to target effector T-lymphocytes whilst sparing Tregs. A recent single-centre randomised control trial demonstrated that the addition of sirolimus to standard GVHD prophylaxis of CSA and MMF led to significantly reduced rates of grade 2 to 4 aGVHD, associated with improved overall survival and non-relapse mortality but no difference in rates of cGVHD in non-myeloablative matched unrelated donor HSCT^19^. The absence of nephrotoxicity in comparison with CNIs makes it an attractive option in patients with renal dysfunction, but there are concerns that sirolimus may increase the risk of endothelial injury and associated thrombotic microangiopathy and veno-occlusive disease^20^. Abatacept, a recombinant CTLA4 fusion protein which interferes with T-lymphocyte co-stimulatory signalling, in addition to standard CNI/MTX-based GVHD prophylaxis, has been shown to be safe and improve rates of aGVHD without increasing the risk of infection or relapse^21^. Similar success with adding abatacept to standard GVHD prophylaxis has been demonstrated in other studies involving patients with non-malignant conditions^22,23^.

**Table 1.** Summary graft-versus-host disease (GVHD) preventative strategies.

GVHD prophylaxis	Outcome
Calcineurin inhibitor (CSA/TAC) plus MTX/MMF (standard GVHD prophylaxis)	Reduces aGVHD and cGVHD
Addition of sirolimus to standard GVHD prophylaxis	Reduces aGVHD but no difference in cGVHD
Addition of abatacept to standard GVHD prophylaxis	Reduces aGVHD
Post-transplant cyclophosphamide	Reduces aGVHD and cGVHD
*In vivo* TCD using ATG	Reduces aGVHD and cGVHD
*In vivo* TCD using alemtuzumab	Reduces aGVHD and cGVHD (increased infection and relapse risk compared with ATG)
*Ex vivo* TCD - CD3+TCRαβ+/CD19+ lymphocyte removal	Reduces aGVHD and cGVHD
*Ex vivo* TCD - removal of naïve T-lymphocytes	Reduces cGVHD but not aGVHD
*Ex vivo* TCD - CD34+ selection with infusion of Tregs (regulatory T-lymphocytes) and conventional T-lymphocytes	Reduces aGVHD and cGVHD

aGVHD, acute graft-versus-host disease; ATG, anti-thymocyte globulin; cGVHD, chronic graft-versus-host disease; CSA, ciclosporin; MMF, mycophenolate mofetil; MTX, methotrexate; TAC, tacrolimus; TCD, T-cell depletion.

Prophylaxis with post-transplant cyclophosphamide (PTCy) has revolutionised the use of haploidentical HSCT in recent years. This strategy has demonstrated low rates of severe aGVHD and cGVHD in haploidentical HSCT with both reduced intensity^24^, and myeloablative conditioning^25,26^, and more recent studies have demonstrated its effectiveness in matched donor HSCT^27,28^. The mechanisms underlying PTCy continue to be investigated but are thought to involve suppression of proliferating alloreactive T-lymphocytes and deletion of intrathymic clonal alloreactive T-lymphocyte precursors whilst sparing Tregs^29^.

*In vivo* T-lymphocyte (cell) depletion (TCD) is another method of reducing GVHD risk, most commonly with anti-thymocyte globulin (ATG), a purified polyclonal immunoglobulin G fraction harvested from sera collected from horses or rabbits immunised with human thymocytes or T-lymphocyte lines. ATG has been shown in several prospective randomised trials to particularly reduce the incidence of cGVHD, although it does not confer improved survival in most studies and is associated with viral reactivation^30–33^. A better understanding of pharmacokinetics and utilisation of therapeutic drug monitoring to allow individualised dosing regimens, particularly for paediatric patients, presents a potential strategy to reduce GVHD risk whilst optimising immune reconstitution^34^. Alemtuzumab, an anti-CD52 monoclonal antibody, is a newer approach to *in vivo* TCD compared with ATG, and comparatively less evidence is available from prospective randomised trials. Its effectiveness in reducing GVHD has been demonstrated, but at the expense of increased infection and relapse risk^35^.

Graft engineering with different *ex vivo* methods of TCD has evolved significantly in recent years. Previous methods include CD34^+^-positive selection and CD3^+^/CD19^+^-negative selection. More recent advances include the successful use of HLA-mismatched grafts in which CD3^+^ T-lymphocytes bearing the TCRαβ receptor and CD19^+^ cells have been selectively removed, and TCRγδ receptor-bearing T-lymphocytes and natural killer (NK) cells have been preserved^36–39^. Other *ex vivo* TCD methods include removal of naïve T-lymphocytes from the graft whilst keeping memory T-lymphocytes, which was shown to reduce cGVHD (though not aGVHD) without impairing engraftment or increasing infection risk^40^, and CD34^+^ selection with an infusion of Tregs and conventional T-lymphocytes on day -4 and D0 of transplant respectively^41^. Another approach under investigation to exploit the potential benefit of Tregs in reducing GVHD is the use of iNKT cells, a population which expresses both T and NK cell markers and possesses immunomodulatory effects via the production of IL-4 and IL-10 and promotes Treg activation and expansion. A 2017 trial using a synthetic derivative of alpha-galactosylceramide, which binds to CD1 leading to iNKT cell activation and expansion, infused on day 0 was well tolerated and was associated with Treg expansion in a subset of patients who had a lower incidence of aGVHD^42^.

## Management

### Immunosuppression/immunomodulation

Corticosteroids remain the first-line treatment option for aGVHD, but only about 50% of patients will respond^43^. The group with non-responsive, progressive or corticosteroid-dependent disease is particularly associated with a poor prognosis^44,45^. The underlying pathophysiology of steroid-refractory GVHD (SR-GVHD) is complex and incompletely understood but may involve a less important ongoing role of donor alloreactive T-lymphocytes than in the initiation of aGVHD^14^. Interference of corticosteroids with Treg-mediated peripheral tolerance, especially when used in combination with agents that disrupt IL-2 pathways^46,47^ and the negative impact of corticosteroids on tissue repair and regeneration leading to ongoing mucosal barrier dysfunction and alterations in the intestinal microbiome^11^, may be more critical. Corticosteroids, along with aGVHD-related thymic damage, impair thymopoiesis, disrupting normal thymic function and delaying the restoration of adaptive T-lymphocyte immunity^48^.

An increasing number of therapeutic options have been investigated for patients with SR-GVHD, although no clear frontrunner has emerged. Extracorporeal photopheresis (ECP) is an apheresis-based therapy involving exposure of mononuclear blood cells to 8-methoxypsoralen and ultraviolet-A radiation, followed by re-infusion of photoactivated cells into the patient. There are no systemic immunosuppressive effects, and ECP facilitates weaning of immunosuppressive medications^49^; therefore, ECP does not increase infection or disease relapse risk. The mechanisms behind ECP have not yet been fully elucidated; it is thought that the ECP procedure induces preferential apoptosis of alloreactive T-lymphocytes, and uptake of apoptotic antigen by APCs precedes multiple processes leading to increased immune tolerance involving dendritic cells, Tregs and changes in cytokine profiles. A small number of ECP-responding patients were shown to display a distinct T-lymphocyte transcriptomic signature with decreased expression of genes important in T-lymphocyte activation, including ERRα and GαS signalling pathways^50^. Recent studies have also reported the possible contribution of immunomodulatory NK cells to the mechanisms behind ECP^51,52^. Confirmation of these findings in additional patients is needed. ECP has repeatedly demonstrated safety and efficacy in SR-aGVHD/cGVHD^49,53,54^ but has often been utilised late in the disease course, and data are limited mainly to small non-randomised studies^55^. A multi-centre phase 3 trial involving paediatric patients with SR-aGVHD demonstrated an overall response rate of 55% at day 28, increasing to 79% by week 12^56^. Previous studies suggested better outcomes with earlier adoption of ECP^54^, and this was observed in a recent phase 2 randomised trial where aGVHD patients who received upfront ECP alongside corticosteroids were more likely to respond compared with those receiving corticosteroids alone, particularly for skin-only aGVHD^57^. However, use of ECP as part of GVHD prophylaxis did not show a benefit in another randomised trial^58^.

Mesenchymal stem cells (MSCs) are another type of cellular therapy with immunomodulatory effects through a diverse range of actions used in both the prevention and treatment of GVHD^59^. Recent meta-analyses evaluating both prevention and treatment of GVHD with MSCs suggest that they may reduce the incidence of cGVHD but not aGVHD. Some studies have shown that MSCs are effective in aGVHD treatment, but the quality of available evidence is low^60,61^. Studies do indicate that MSCs are safe and well tolerated. There are ongoing questions regarding the optimal timing of MSC infusion, and more robust data in the form of well-designed clinical trials are required to confirm their efficacy.

JAK signalling plays an integral role in the pathogenesis of aGVHD, including the production of inflammatory cytokines leading to activation of APCs and subsequent activation and migration of alloreactive T-lymphocytes. The use of ruxolitinib, a JAK1/2 inhibitor, demonstrated an overall response rate of 62% in patients with SR-aGVHD in the REACH2 trial, a multi-centre randomised trial comparing ruxolitinib with the best available therapy^62^. Side effects include cytopaenias and increased infection risk, but there does not appear to be a negative impact on the graft-versus-leukaemia effect^9^. In the REACH3 randomised trial evaluating ruxolitinib compared with best available therapy for SR-cGVHD, the 6-month overall response rate was superior in the ruxolitinib group (49.7% vs. 25.6%), although complete response rates were low in both groups (6.7% vs. 3.0% respectively), highlighting the difficulty in successfully treating these conditions^63^. Other early promising data are emerging on selective targeting of JAK1 with itacitinib^64^ and inhibition of spleen tyrosine kinase^65^.

Evidence implicating the role of dysregulated B-lymphocyte immunity in cGVHD has prompted the development of B-lymphocyte-directed therapies such as the anti-CD20 monoclonal antibody rituximab, which has shown efficacy in corticosteroid-refractory disease^66^ and as first-line therapy^67^. Tyrosine kinase inhibitors (TKIs) are another group of emerging therapies in cGVHD. Ibrutinib is a TKI with inhibitory effects on Bruton’s tyrosine kinase and interleukin-2 inducible T-cell kinase (ITK), consequently blocking B- and T-lymphocyte signalling and activation, respectively, and significant promise has been observed in preclinical studies^68,69^. Initial clinical data include a study in which 71% of patients demonstrated a sustained response at a median follow-up of 13.9 months^70^. Further clinical studies include the iNTEGRATE phase 3 clinical trial investigating upfront ibrutinib in combination with corticosteroids in moderate to severe cGVHD (ClinicalTrials.gov identifier: NCT02959944) and an ongoing clinical trial investigating ibrutinib as a first-line solo therapy in cGVHD (ClinicalTrials.gov identifier: NCT04294641).

Other new strategies for cGVHD include targeting the ROCK2 (rho-associated coiled-coil–containing protein kinase-2) signalling pathway, which regulates Th17/regulatory T-lymphocytes. Inhibition of ROCK2 signalling reduces Th17 lymphocytes, promotes Tregs and has an anti-fibrotic effect^71^. In a phase 2 clinical trial, belumosudil, an oral ROCK2 inhibitor, demonstrated an overall response rate of 62% to 69% depending on the dose used, was associated with improved quality of life and weaning of corticosteroids and was well tolerated^72^. Another randomised multi-centre trial demonstrated an overall response rate of 77%, a sustained (median of 54 weeks) response, and improvement in all organ systems affected^73^.

Novel approaches on the horizon for managing SR-GVHD include a combination of anti-CD3 and anti-CD7 antibodies conjugated to recombinant ricin A, which induces *in vivo* T- and NK cell depletion and suppresses T-lymphocyte receptor activation. A phase 1/2 trial involving 20 patients with SR-aGVHD demonstrated a day 28 overall response rate of 60% and a complete response rate of 50%^74^, and further investigation in a phase 3 trial is in progress (CTN 1802). Other new T-lymphocyte-directed therapies include brentuximab vedotin (targeting CD30 expression on activated CD8^+^ T-lymphocytes in aGVHD)^75^ and vedolizumab (anti-α4β7 integrin that inhibits T-lymphocyte migration)^76^. CD6 is predominantly expressed on T-lymphocytes and binds to activated leukocyte cell adhesion molecule (ALCAM) expressed on APCs and various host tissues and plays an integral role in T-lymphocyte activation, proliferation, differentiation and migration. A phase 1/2 clinical trial (ClinicalTrials.gov identifier: NCT03763318) is evaluating itolizumab, a humanized anti-CD6 monoclonal antibody, as first-line treatment for aGVHD alongside corticosteroids. Finally, another novel intervention for GVHD undergoing investigation is faecal transplantation. Disruption of the intestinal microbiome is associated with an increased risk of aGVHD, and restoration of the gut with normal diverse gut flora has been shown to be safe and effective in gastrointestinal SR-aGVHD in some early studies^77–79^.

## Tissue protection/regeneration

Based on recent studies suggesting impaired tissue repair mechanisms in SR-aGVHD, alternative approaches in development target tissue protection and promote recovery following insult. Alpha-1 antitrypsin is a protease inhibitor that protects tissues from proteolytic degradation but, in aGVHD murine models, has also been shown to reduce pro-inflammatory cytokines and increase Tregs and, in a clinical trial, was demonstrated to be safe and effective in SR-aGVHD (overall response rate of 65% at day 28) and have low rates of infection^80^. Other tissue-supportive strategies include epidermal growth factor derived from urinary-derived human chorionic gonadotropin^81^, lithium^82^, glucagon-like-peptide-2^83^ and interleukin-22^84^.

## Risk-adapted approach and biomarkers

A challenge to physicians is predicting the trajectory of the GVHD course to allow a risk-stratified management approach and pre-emptive therapy for individual patients to improve outcomes. This would allow the reduction of treatment in those with mild disease to lessen the risk of treatment-associated adverse effects and early intensification of treatment in patients projected to have high-risk disease. A risk-adapted approach can utilise clinical factors such as the Minnesota Risk Score for aGVHD^85^ and the Hematopoietic Cell Transplantation-specific Comorbidity Index (HCT-CI)^86^ or blood biomarkers. Identification of reliable biomarkers in aGVHD and cGVHD would be instrumental in allowing risk stratification. Many candidate biomarkers have been investigated to date, but at present, none is widely used in clinical practice, and this is due mainly to a lack of validation in large prospective clinical trials^87^. Promising biomarkers evaluated in recent studies include suppressor of tumorigenicity-2 (ST2) and regenerating islet-derived protein 3-α (REG3α) in aGVHD^88,89^. The Mount Sinai Acute GVHD International Consortium (MAGIC) algorithm uses these two biomarkers to predict mortality in aGVHD and has the potential to implement early risk-stratified therapies^90^. Readily available markers indicating endothelial injury (lactate dehydrogenase, serum creatinine and platelet count) have shown promise in predicting GVHD outcomes (Endothelial Activation and Stress Index, EASIX)^91^. Serum albumin at GVHD onset has been shown to identify those with severe disease as well as correlate with prognosis in those with SR-aGVHD^92,93^. Amphiregulin, an epidermal growth factor receptor ligand, has also been used to stratify patients into high-risk subgroups in aGVHD^94^. Promising biomarkers in cGVHD include CXCL9, CXCL10, sBAFF, ST2 MMP3, osteopontin, CD163, IL-17A and IL-21^95^. Significant morbidity and mortality are associated with long-term high-dose corticosteroids, and future biomarker-guided clinical trials must aim to substitute or reduce corticosteroids by, for example, identifying those who would benefit from alternative upfront therapies or patients who would tolerate a rapid corticosteroid tapering approach.

## Summary

Despite significant advances, aGVHD and cGVHD continue to be significant challenges and causes of HSCT-related morbidity and mortality, particularly for patients with corticosteroid-refractory disease. Even when GVHD is controlled by corticosteroids, many patients have adverse side effects and die from infections related to immunosuppression. Barriers to progression in managing patients with SR-GVHD include the inability to accurately risk-stratify, an incomplete understanding of the pathophysiology behind corticosteroid resistance, a deficiency of high-quality evidence to determine optimal treatment strategies, and the difficulties in comparing strategies because of heterogeneity in many factors, including patients, donors and conditioning regimens. Ongoing efforts to improve our understanding of GVHD pathophysiology, development of targeted therapies, further prospective randomised trials to provide more robust data and validated biomarkers, and collaboration between centres will help to inform physicians of optimal GVHD prophylactic and therapeutic strategies and ultimately allow an individualised precision-prevention and treatment approach.
